# White matter hyperintensities volume and cognition: A meta-analysis

**DOI:** 10.3389/fnagi.2022.949763

**Published:** 2022-09-01

**Authors:** Wenjuan Guo, Jing Shi

**Affiliations:** The 3rd Department of Neurology, Dongzhimen Hospital, Beijing University of Chinese Medicine, Beijing, China

**Keywords:** WMH, cognition, dementia, cerebral small vessel disease, meta

## Abstract

**Background:**

Cerebral small vessel disease (CSVD) is prevalent in the elderly and leads to an increased risk of cognitive impairment and dementia. The volume of white matter hyperintensities (WMHs) increases with age, which affects cognition.

**Objective:**

To explore the relationship between WMH volume and cognitive decline in patients with CSVD.

**Methods:**

We performed a systematic search of PubMed, Embase, and the Web of Science databases from their respective creation dates to the 5 May 2022 to identify all the clinical studies on either mild cognitive impairment (MCI) or dementia in regards to WMH volume in CSVD.

**Results:**

White matter hyperintensities was associated with the risk of both the MCI and dementia, with a 35% increased risk [relative risk (RR) = 1.35; (95% CI: 1.01–1.81)] of progression from cognitively unimpaired (CU) to MCI (six studies, *n* = 2,278) and a 49% increased risk [RR = 1.49; (95% CI: 1.21–1.84)] of progression to dementia (six studies, *n* = 6,330). In a subgroup analysis, a follow-up period of over 5 years increased the risk of MCI by 40% [RR = 1.40; (95% CI: 1.07–1.82)] and dementia by 48% [RR = 1.48; (95% CI: 1.15–1.92)].

**Conclusion:**

White matter hyperintensities was found to be substantially correlated with the risk of cognitive impairment. Furthermore, cognitive decline was found to be a chronic process, such that WMH predicted the rate of cognitive decline in CSVD beyond 5 years. The cognitive decline observed in patients with WMH may, therefore, be minimized by early intervention.

## Introduction

Cerebrovascular small vessel disease (CSVD) is an age-related microvascular disease affecting the cerebral small arteries, small veins, and capillaries. The incidence of CSVD is related to the slow accumulation of tissue damage, which primarily manifests as static focal cerebrovascular diseases such as static lacunar infarction (LI), cerebral microbleeds (CMBs), local leukoaraiosis, and progressive cognitive dysfunction (Zhang et al., 2010), which are the leading causes of both the cognitive impairment and vascular dementia (VD) (Cipollini et al., 2019). The identification of CSVD most commonly relies on MRI markers, including white matter hyperintensities (WMHs), LI, enlarged perivascular spaces (EPVSs), CMBs, and brain atrophy (Pantoni, 2010), all of which have variably been associated with cognitive performance (Chojdak-Łukasiewicz et al., 2021). White matter is comprised of the structural connections between areas of the brain's gray matter. These white matter tracts carry information within the brain to sustain a variety of cognitive functions. Consequently, if white matter is disrupted, information transmission is disrupted. Studies have shown that a larger WMH volume is associated with slower local network transfer efficiency and information processing speed (Hilal et al., 2021). Furthermore, in the white matter tracts, which are important for information processing, having a greater WMH load was associated with slower cognitive speed (Vergoossen et al., 2021). Therefore, this study intended to investigate the effect of WMH volume on cognitive function. By systematically reviewing the existing literature, we sought to identify the short- and long-term incidence of WMH volume in subjects with CSVD and its association with cognitive impairment and dementia.

This was a quantitative analysis of the relationship between brain WMH volume and cognition in CSVD focusing on cohort studies that screened patients with CSVD (specifically nondementia, or other diseases that cause high signal changes in white matter, such as nonmajor vascular stroke or multiple sclerosis). We analyzed data from 14 studies on the impact of brain WMH volume on the risk of cognitive impairment to investigate whether there exists a link between WMH volume and cognitive impairment in CSVD.

## Methods

### Study selection criteria

This meta-analysis was conducted on literature obtained from the databases such as PubMed, Embase, and the Web of Science. To assess the interactions between WMH volume and cognition, the literature search was conducted using the keywords such as “cognition,” “dementias,” and “mild cognitive impairment” in combination with either “white matter hyperintensities volume” or “WMH.” We searched all the entries published up until 5 May 2022. All the searches were conducted by combining the Medical Subject Headings (MeSHs) terms and free text. We also screened any relevant references, including related review articles that were referred to in each publication.

### Inclusion criteria

We included both the observational cohort and case–control studies. The included studies determined brain WMH by MRI and white matter hyperintensity volume was calculated by FSL, MATLAB, or other computer segmentation software. Patients were diagnosed as either cognitively unimpaired (CU) or mild cognitive impairment (MCI) based on both the clinical assessment of their functional status in daily life and neuropsychological testing. Participants were excluded if they had a history of dementia, stroke, or other neurological problems. For the analysis of the prevalence of cognitive impairment and dementia in patients with a high white matter signal in the context of CSVD, articles with unclear baseline cognitive levels, i.e., studies without a clear “CU” or “MCI” at baseline, were excluded. There were no differences in either the gender distribution or education level between groups. Reported odds ratios (ORs), relative risks (RRs), and hazard ratios (HRs) were covered, and literature containing only the correlation coefficient values β and SE was excluded. Additionally, this study excluded case reports, cross-sectional studies, animal studies, and reviews that did not contain the original data.

### Data extraction and synthesis

The following data were extracted: authors, year of publication, sample size, study design, duration of follow-up, patient source, outcome measures, odds ratio (OR), relative risk (RR), hazards ratio (HR), and primary data. Effect estimates adjusted for covariates such as age, sex, and education were analyzed. If no OR, RR, or HR was reported, they were calculated from the original data. Studies were excluded if neither the raw data nor the OR/RR/HR was provided.

The RR and 95% CI were calculated using the R 4.2.0 “meta” package to summarize the RR/OR/HR. The total pooled effects were based on estimating cohort-specific effects. Heterogeneity across estimates was evaluated by the Cochran's *Q* statistic [a *P*-value assessed using *I*^2^ (*P*-value < 0.05 and *I*^2^ > 50% were considered to be indicative of statistically significant)]. If there was little evidence of heterogeneity between studies (i.e., from Cochran's *I*^2^ statistics < 50%), the fixed effects models were used to pool effect estimates. If there was heterogeneity between studies, we used the random effects models and probed for sources of heterogeneity through sensitivity analysis. Subgroup analyses were performed in accordance with the baseline cognitive level of subjects and their follow-up progression of cognitive impairment. We assessed publication bias using Egger's test. If statistically significant publication bias was found, the “trim-and-fill” method was utilized to adjust for the bias. The methods and results reported in this meta-analysis have been based on the Preferred Reporting Items for Systematic Reviews and Meta-Analyses (PRISMA) (Moher et al., 2009) guidelines. The nine star Newcastle–Ottawa Scale (NOS; accessed *via* http://www.ncbi.nlm.nih.gov/books/NBK35156/) (Stang, 2010) was used to appraise the quality of the cohort studies based on three dimensions: the selection of study subjects, the comparability of exposure groups, and the measurements of the outcomes of interest. Studies rated nine stars were considered high-quality studies. Studies rated either seven or eight stars were considered to be of moderately high quality. A rating below seven stars was deemed to be low quality.

## Results

**Figure 1:**
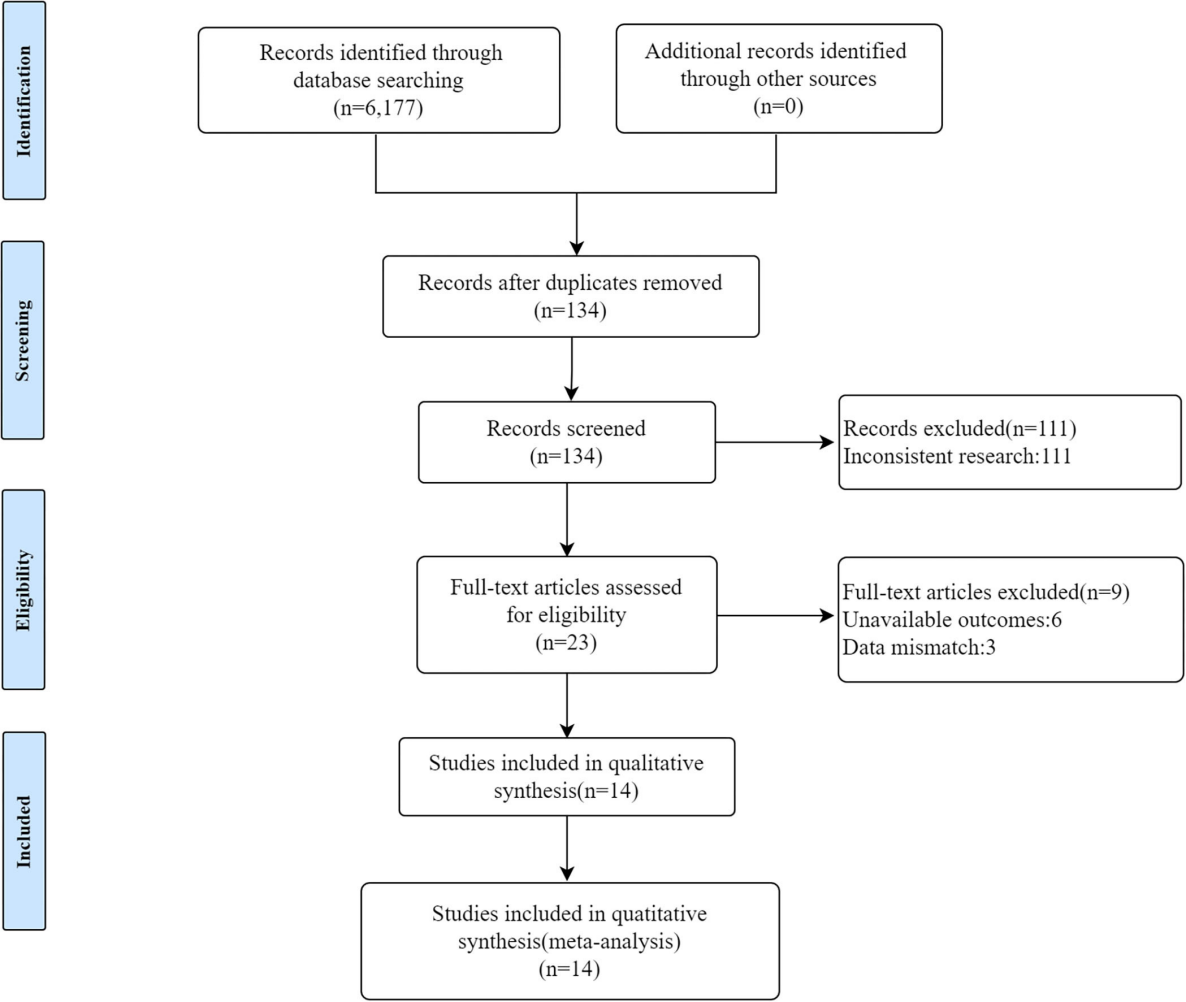
Study flow chart.

A total of 6,177 literature entries from PubMed, Embase, and the Web of Science were retrieved. A total of 134 reports remained after the initial screening by removing duplicate literature items, old literature, reviews, animal experiments, etc., that failed to meet the inclusion criteria. Finally, leaving 14 items of literature are to be included for full-text reading and analysis (Figure 1). Six of these 14 studies were considered high quality, while six studies were considered medium-quality articles (Table 1).

**Table 1:** Newcastle-Ottawa scale.

**Study ID**	**Represent ativeness of the exposed cohort**	**Selection of the non-exposed cohort**	**Ascertainment of exposure**	**Outcomes of interest does not appear at start of study**	**Study controls for the most important factor**	**Study controls for any additional factor**	**Assessment of outcome**	**Was follow-up long enough for outcomes to occur**	**Adequacy of follow up of cohorts**
Xia et al. (2020)	*	*	*	*	*	*	*	*	*
Müller et al. (2020)	*	*	*	*	/	*	*	*	*
Burke et al. (2019)	*	*	*	/	*	*	*	/	*
Xiong et al. (2018)	*	*	*	/	/	/	*	*	*
Lopez et al. (2018)	*	*	*	/	/	/	*	*	*
Bangen et al. (2018)	*	*	*	/	*	*	*	*	*
Boyle et al. (2016)	*	*	*	*	*	*	*	*	*
van Uden et al. (2016)	*	*	*	*	*	*	*	*	*
Weinstein et al. (2013)	*	*	*	*	*	*	*	*	*
Godin et al. (2010)	*	*	*	/	*	*	*	*	*
Debette et al. (2010)	*	*	*	/	*	*	*	*	*
Ikram et al. (2010)	*	*	*	*	*	*	*	*	*
Silbert et al. (2009)	*	*	*	*	*	*	*	*	*
Smith et al. (2008)	*	*	*	/	/	*	*	*	*

*The study meets this methodological description.

A total of 10,444 enrolled patients participated. It was determined that 999 of the 10,444 (10%) participants developed a change in their cognitive diagnosis from baseline to follow-up. Of these patients, 7,474 patients were CU at baseline, 367 patients were patients with MCI, and 2,603 patients were CU- and MCI-promiscuous enrollees. Among them, 335 (4.48%) participants had incident MCI, while 368 (4.92%) participants had incident dementia (NAD = 211, AD = 157), the latter of which was comprised of 189 patients with CU, 103 patients with MCI, and 76 patients with CU + MCI.

The 14 cognition-related studies differed in their approaches used for cognitive testing. Nine studies used the Mini-Mental State Examination (MMSE), while four studies applied the Clinical Dementia Rating (CDR; Table 2). Cognitive function was assessed in all the participants at both the baseline and the end of follow-up.

**Table 2:** Characteristics of included studies.

**Study ID**	**Country**	**Study design**	**Total patients consented/total tested**	**Follow up**	**MRI method**	**Test done**	**Adjustment**
Xia et al. (2020)	China	Cohort Study	MCI: 191/41	7 years	1.5-Tesla GE/3.0-Tesla GE MRI scanner T1-WI, T2-WI, FLAIR	MMSE AVLT HOMT COST TMT	Age, sex, interval, education, ApoE e4 carrier
Müller et al. (2020)	Swedish	Cohort Study	Dementia: 212/16	6 years	1.5T MRI scanner (Philips Intera, The Netherlands) T1-WI, T2-WI, FLAIR	MMSE	Age, sex, and education
Burke et al. (2019)	USA	Cohort Study	MCI: 483/45 Dementia: 211/49	3 years	T1-WI, T2-WI, FLAIR	CDR	Age, CDR global score, and neuropsychiatric symptoms, White non-Hispanic race and CDR global score
Xiong et al. (2018)	China	Cohort Study	Dementia: 158/53	14 years	1.5 T MR scan (Siemens Healthcare, Magnetom Avanto, Erlange, Germany) T1-WI, T2-WI, FLAIR	BDS CDR	/
Lopez et al. (2018)	USA	Cohort Study	Dementia: 183/23	5.7 years	GE Signa 1.5T scanner T1-WI, T2-WI, FLAIR	MMSE	/
Bangen et al. (2018)	USA	Cohort Study	MCI: 561/72	6.5 years	1 or 1.5 Tesla Siemens Magnetom MRI scanner T1-WI, T2-WI	WMS TMT BNT	Age at MRI, sex, days between baseline MRI and baseline neuropsychological assessment, education, APOE ε4 status, vascular risk factors, other MRI measures
Boyle et al. (2016)	USA	Cohort Study	MCI: 354/106	6 years	1.5 Tesla General Electric (Waukesha, WI) MRI scanner T1-WI, T2-WI, FLAIR	MMSE	Age, gender, and education Total gray matter vascular risk factors and diseases
van Uden et al. (2016)	Holland	Cohort Study	Dementia: 500/42	5.2 years	1.5-Tesla MRI (Magnetom Sonata, Siemens Medical Solutions, Erlangen, Germany) T1-WI, T2*-WI, FLAIR,DTI	MMSE	Age, gender, education, baseline MMSE and territorial infarcts
Weinstein et al. (2013)	USA	Cohort Study	Dementia: 1,414/28	10 years	1 or 1.5-Tesla Siemens Magnetom scanner 3D T1, PD, T2	LM VR PAS SIM HVOT TrA TrB BNT DSF DSB COWAT	Age, sex, education, hypertension, current smoking, history of diabetes mellitus, body mass index, and ApoEε4 status
Godin et al. (2010)	France	Cohort Study	MCI: 1,701/224 Dementia: 1,701/46	4 years	1.5 Tesla Magnetom (Siemens, Erlangen) MRI scanner T1-WI, T2-WI	MMSE Tr B IST BVRT	Sex, age, education level, hypertension, history ofcardiovascular disease, diabetes, MMSE score, hypercholesterolemia and ApoE genotype and TIV
Debette et al. (2010)	USA	Cohort Study	MCI: 1,134/93 Dementia: 2,013/11	6 years	1 or 1.5-T Siemens Magnetom T1, T2	NP	Age, gender systolic blood pressure, current smoking, diabetes, history of cerebrovascular disease, interim stroke, excluding prevalent stroke (for dementia) or for interim stroke and dementia and excluding prevalent stroke and dementia (for death)
Ikram et al. (2010)	Holland	Cohort Study	Dementia: 490/46	5.9 years	1.5-T MRI System (VISION MR, Siemens AG, Erlangen, Germany) T2WI, 3D HASTE	MMSE Letter-Digit Substitution Task the Stroop test verbal fluency task 15 word verbal learning test	Age, sex, education diabetes mellitus, smoking, hypertension and brain infarcts
Silbert et al. (2009)	USA	Cohort Study	MCI: 49/24	5.6 years	1.5-T magnet T1-WI, T2-WI	MMSE CDR	Age, incident HTN, ICV, MMSE at entry, baseline hippocampal and vCSF volume, ApoE-4 status, and baseline MRI volume
Smith et al. (2008)	USA	Cohort Study	MCI: 67/26 Dementia: 156/54	6 years	1.5-T scanners T2-WI	CDR MMSE	Age, sex,education, past smoking, and APOE genotype

MMSE, mini-mental state examination; MCI, mild cognitive impairment; AVLT, auditory verbal learning test; COST, common objects sorting test; TMT, trail making test; CDR, clinical dementia rating; NP, neuropsychological test battery; BDS, blessed dementia scale; WMS, Wechsler Memory Scale; LM, logical memory; VR, visual memory; PAS, paired associate; SIM, immediate recall and similarities; HVOT, Hooper visual organization test; TrA, trail making test A; TrB, trail making test B; BNT, Boston naming test; DSF, digit span forward; DSB, digit span backward; COWAT, controlled word association fluency test; IST, Isaacs' set test; BVRT, Benton visual retention test; T1-WI, T1 weighted images; T2-WI, T2 weighted images; FLAIR, fluid attenuation inversion recovery; PD, double echo proton density; TIV, total intracranial volume; ICV, intracranial volume; vCSF, ventricular cerebral spinal fluid; HTN, hypertension.

According to the time of follow-up, we divided the included literature into two subgroups: more than 5 years and <5 years (Table 2). Studies with new-onset MCI had a mean follow-up time of 5.6 years. The mean follow-up time for the progression from baseline CU to dementia was 6.2 years, and the mean follow-up time for the conversion from baseline MCI to dementia was 4.5 years.

**Figure 2:**
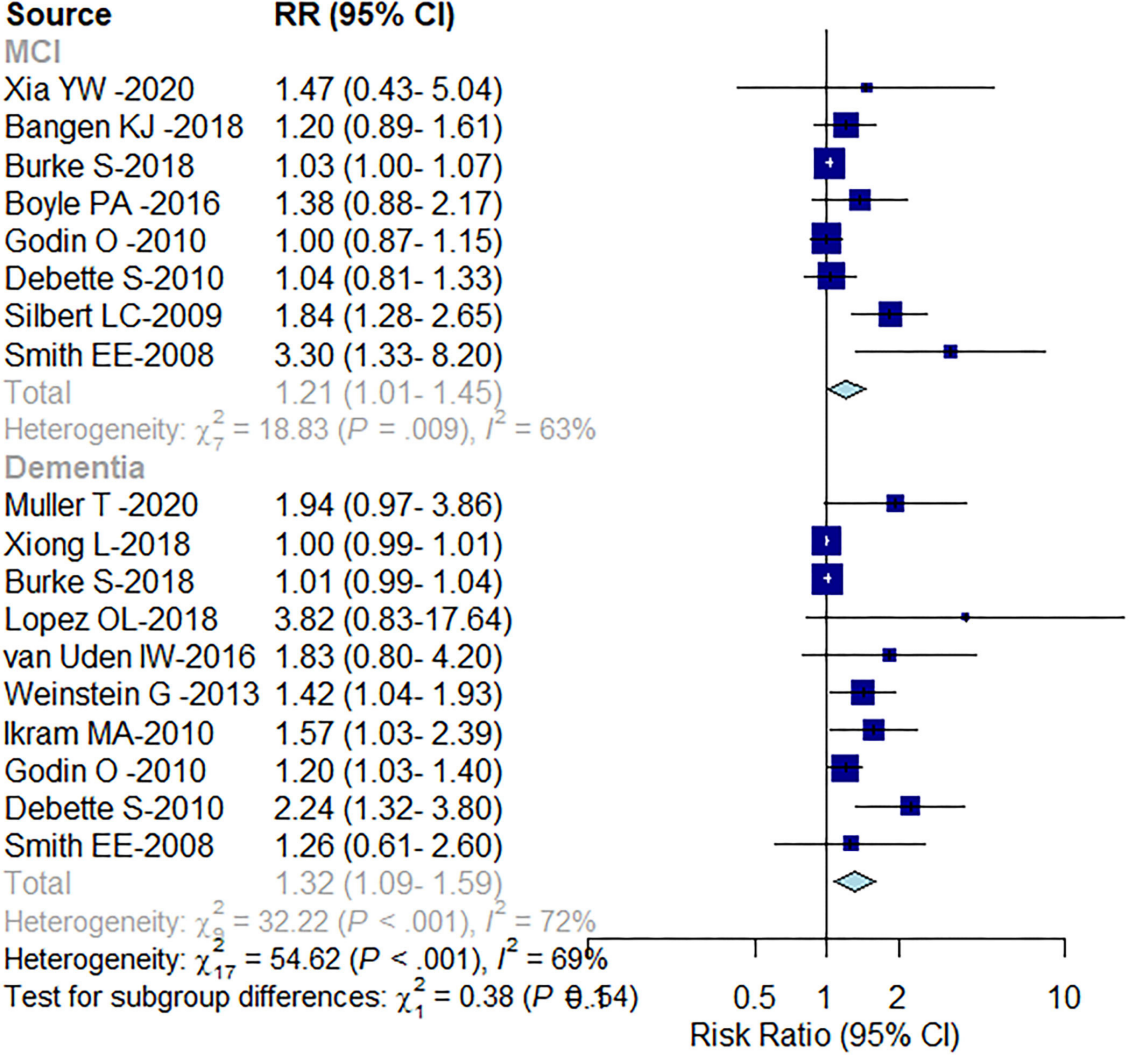
Forest plot of the correlation between WMH volume and cognition.

**Figure 3:**
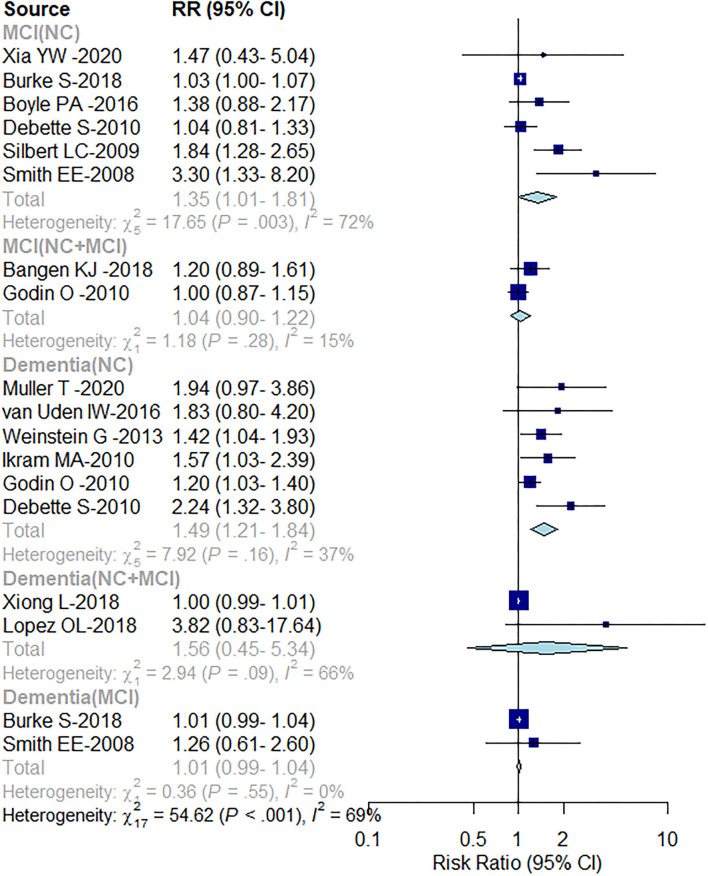
Subgroup analysis based on cognitive level.

WMH was found to increase the risk of both the MCI and dementia. The cognitive score-based analysis conducted in the present study showed an association between WMH and both the MCI and dementia, where the risk of MCI was 21% greater [RR = 1.21; (95% CI: 1.01–1.45), *P* = 0.009], and that of dementia was 32% greater [RR = 1.32; (95% CI: 1.09–1.59), *P* < 0.001; Figure 2]. There was, however, marked heterogeneity between the studies (MCI: *I*^2^ = 63%; dementia: *I*^2^ = 72%). Subgroup analysis by the baseline level of cognition showed a 35% [RR = 1.35; (95% CI: 1.01–1.81)] increased risk of progression from CU to MCI and risk of dementia by 49% [RR = 1.49; (95% CI: 1.21–1.84)], but no increased risk of progression of dementia in patients with MCI [RR = 1.01; (95% CI: 0.99–1.04); Figure 3].

**Figure 4:**
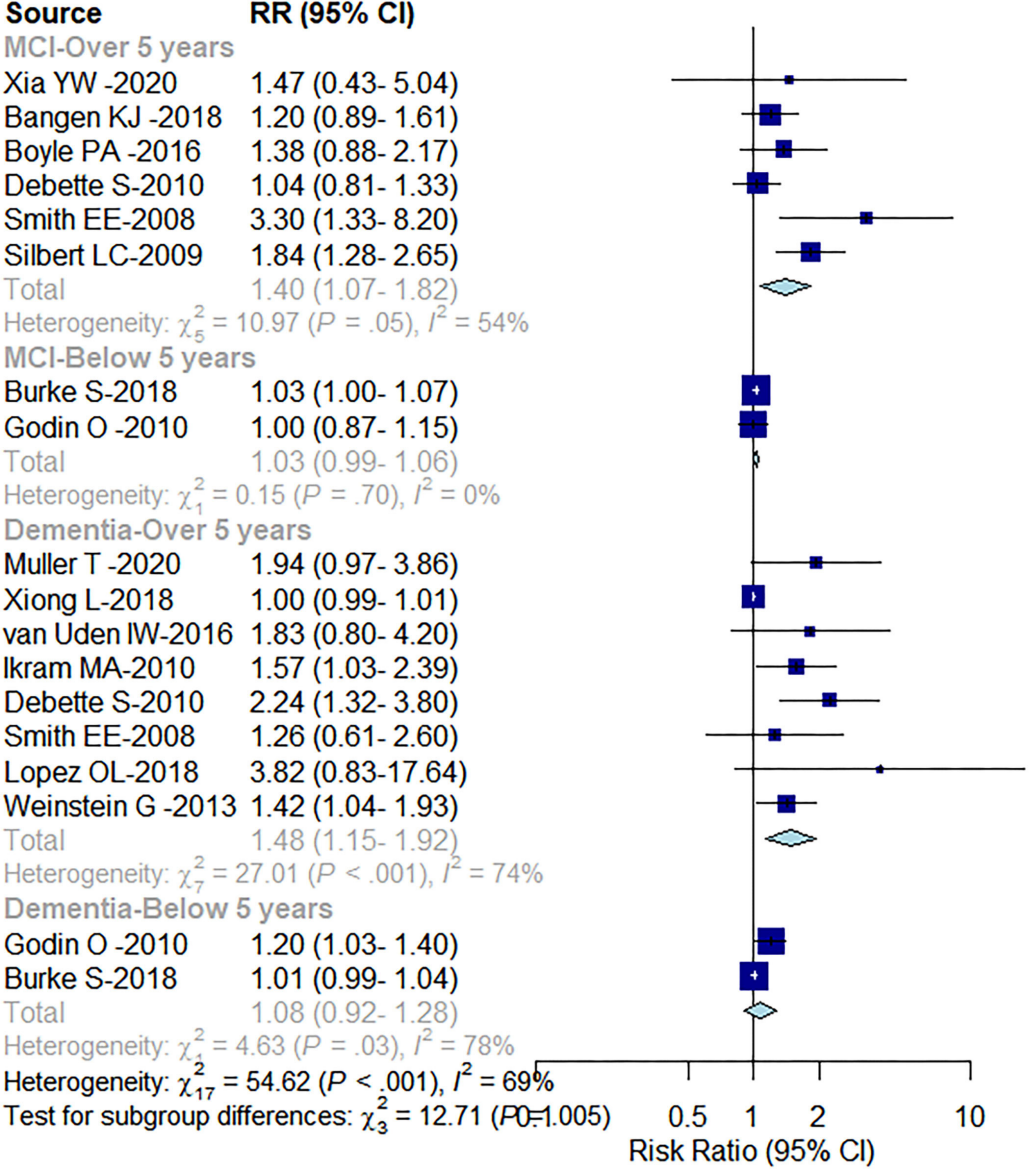
Subgroup analysis based on follow-up time.

In a subgroup analysis by the duration of follow-up, patients with >5 years of follow-up showed a higher risk of developing MCI and dementia than patients with a follow-up period of fewer than 5 years. A follow-up period of over 5 years increased the risk of MCI by 40% [RR = 1.40; (95% CI: 1.07–1.82)] and dementia by 48% [RR = 1.48; (95% CI: 1.15–1.92)]. No association between MCI and dementia progression was observed in subgroups with less than a 5-year period of follow-up [MCI: RR = 1.03; (95% CI: 0.99–1.06); dementia: RR = 1.08; (95% CI: 0.92–1.28); Figure 4].

**Figure 5:**
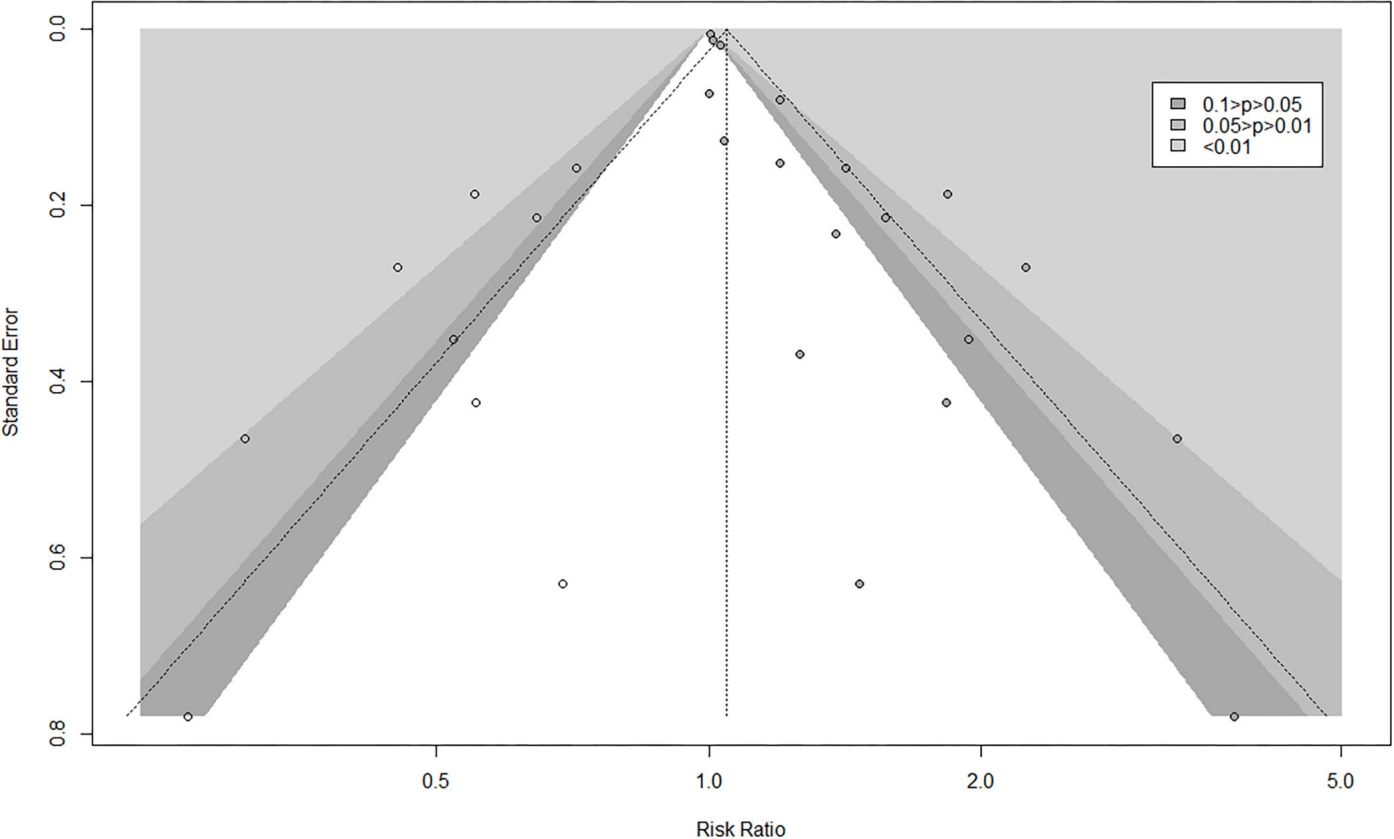
Publication bias by trim-and-fill.

**Figure 6:**
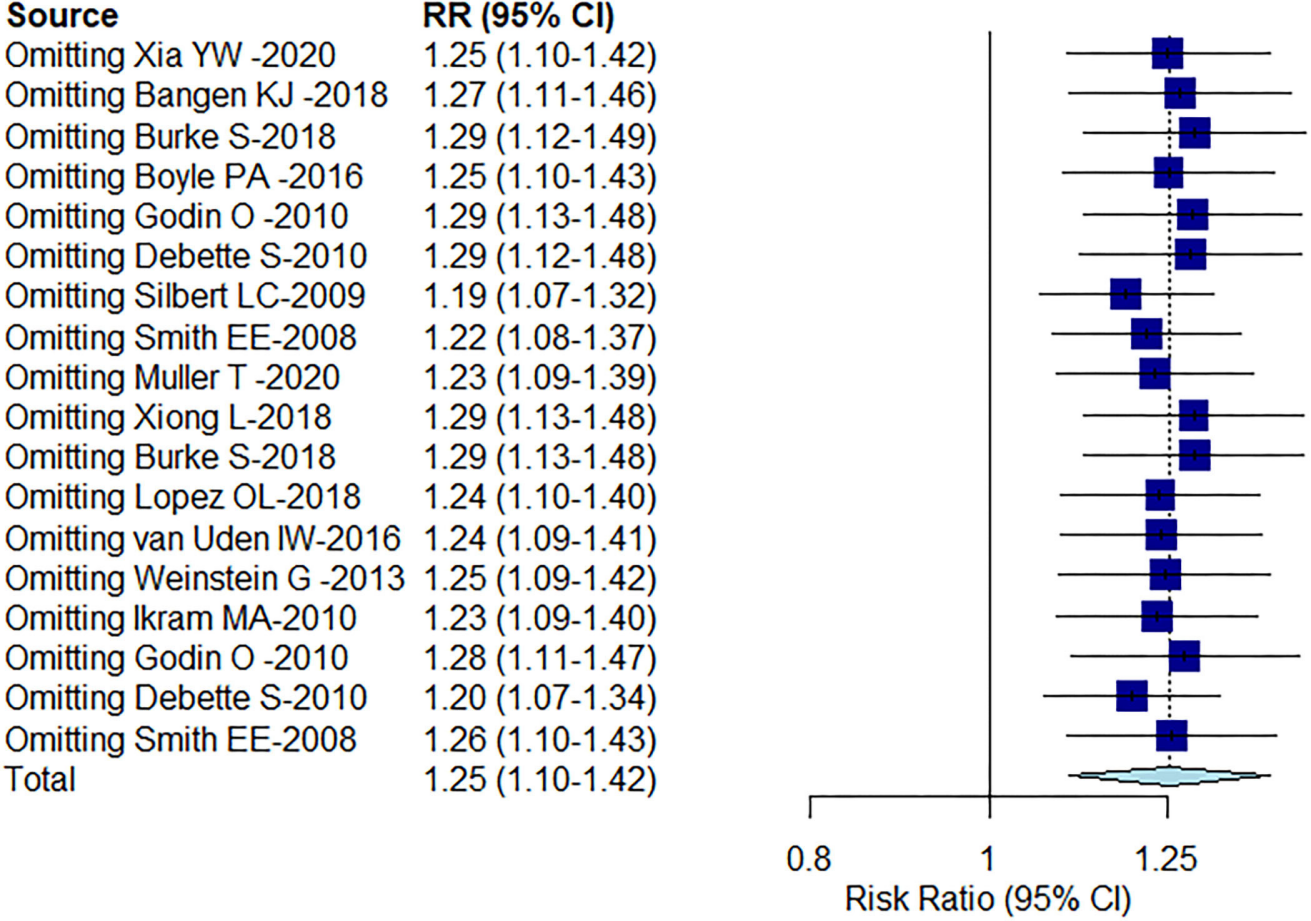
Sensitivity analysis of the included literature on the correlation between WMH volume and cognition.

The result of Egger's test detected publication bias (*t* = 6.94, *df* = 16, *P*-value < 0.0001). This was corrected by using the “trim-and-fill” method (Figure 5), and the effect size after simple compliment was RR = 1.0475 (95% CI: 0.8742–1.2551). Observably, the funnel plot is approximately symmetrical, and there was a certain publication bias, but it had no substantial impact on the overall results. Sensitivity analysis found no outliers (Figure 6). However, the progression of the disease was protracted; only 16% (290/1,795) of participants progressed to an MCI diagnosis after a mean follow-up of 6 years, while 3% (143/4,629) of participants progressed from CU to dementia.

## Discussion

The types of brain markers associated with CSVD include WMH, LI, EPVS, CMB, and brain atrophy (Heiland et al., 2021). CSVD is common in older adults (Kim et al., 2018) and is an important cause of cognitive and motor impairments (Willey et al., 2013). Studies have found that the overall volume of WMH increases with age (Franchetti et al., 2020), and the presumed mechanism behind this is that CSVD disrupts the structural connectivity of large-scale brain networks, thereby impairing the ability of the brain to efficiently integrate neural processes (Du et al., 2020). Some studies have suggested that WMH interferes with these brain networks by either directly or indirectly reducing brain region-specific functional connectivity (Langen et al., 2018). Various imaging and clinical studies have shown that white matter damage is closely associated with cognitive impairment and can increase the prevalence of neuropsychiatric disorders (Clancy et al., 2021), such as major depressive disorder, bipolar disorder, and schizophrenia. Studies have also shown that the effect of WMH on brain function varies according to its localization (Portet et al., 2012). For example, periventricular WMH (PVWMH) and deep WMH (DWMH) have distinct functional and clinical relevance. PVWMH is associated with impaired cognitive function, especially processing speed and executive function (Debette and Markus, 2010; Griffanti et al., 2018), while DWMH is associated with impairments in basic activities of daily living (Park et al., 2022).

This meta-analysis included 14 studies comprised of a total of 10,444 participants. Conjoint analysis showed that WMH volume was tied to the risk of cognitive impairment. To the best of our knowledge, this is the first meta-analysis conducted to investigate the association between WMH volume and the risk of cognitive impairment using solely longitudinal studies. Previous studies, including case–control studies and cohort studies, have explored the association of WMH with cognitive or neuropsychiatric symptoms, and in addition to inconsistent consequences, the underlying disease in patients with WMH has been uniform. For example, one study (Georgakis et al., 2019) reported the existence of a relationship between WMH and cognitive impairment after ischemic stroke. Clancy et al. (2021) reported the relationship between WMH and neuropsychiatric symptoms in CSVD. Through a further review of the existing cohort studies, most have suggested that WMH volume is associated with cognitive changes (Müller et al., 2020; Xia et al., 2020). In our study, the heterogeneity analysis showed high heterogeneity, so we combined an adjusted or a random effects model. In addition, both the cognitive decline and WMH in CSVD are age-related, and white matter loss accelerated with age (Smith et al., 2015). Therefore, longer follow-up time also represented an increase in patient age and white matter damage. However, follow-up time varied greatly among participants included in the study. To identify the sources of heterogeneity, we performed subgroup analyses of participants, cognitive levels at baseline, and the duration of follow-up across studies. Despite the presence of publication bias, by employing the “trim-fill” method, we determined that publication bias was unlikely to have had a significant effect on the results. The sensitivity analysis results were stable. Our meta-analysis did not include extra diseases that are known to cause WMH (such as Parkinson's disease, Alzheimer's disease, cerebrovascular disease, multiple sclerosis, and other neurological diseases) because they have a different clinical pathogenesis.

This article has some limitations. There exist different etiologies and pathogenic outcomes of WMH situated in different regions within the brain (Annweiler and Montero-Odasso, 2012); however, only two studies in the included literature dealt with WMH distribution. van den Heuvel et al. (2006) suggested that the PVWMH volume at baseline is longitudinally associated with reduced cognitive processing speed, which was shown in a cohort-based study. Another study by Godin et al. (2010) concluded that PVWMH was slightly more highly correlated with severe cognitive deterioration compared to DWMH. Unfortunately, we were unable to perform regional subgroup analysis on this topic due to a lack of sufficient data. Based on the available data, we could only perform a summary analysis of the total WMH volume. The relationship between different regions (DWMH, PVWMH) and cognition, therefore, requires more data for further analysis. Furthermore, the methods and software algorithms used for measuring the volume of WMH in our included studies were not consistent, which may have influenced the results. Future studies should, therefore, employ a consistent approach to determine the characteristic manifestations of WMH volume.

A second problem with the data used in this study is the small number of studies included in our analysis. There were only two studies involving patients with MCI at baseline. This reduced the power of our statistical analysis on the risk of developing dementia from MCI, as well as introduced uncertainty to the results. In this meta-analysis, the necessary exclusion criteria exacerbated the results of the small number of studies; for example, our meta-analysis excluded other disorders that cause WMH (such as Parkinson's disease, Alzheimer's disease, cerebrovascular disease, and other neurological diseases such as multiple sclerosis) because they have different clinical pathogenesis. Additionally, different methods used for reporting results were utilized between studies; some studies provided raw data from which OR, RR, or HR could be calculated, while other studies provided adjusted or unadjusted OR/RR/HR or other forms of statistical measurements. This variability created analytical compatibility issues and prevented some studies from being enrolled in the analysis due to a lack of obtainable data. Additionally, some studies controlled for covariates, while other studies that performed multivariate analyses differed in the covariates included, thus affecting this meta-analysis. Moreover, patients with other imaging markers of CSVD and WMH should be examined separately to determine their respective specific impacts on cognitive changes. This meta-analysis suggests that WMH volume increases the likelihood of cognitive alterations. However, heterogeneity between the included studies and patient populations, the small number of studies analyzed, and the differences in measurement methods prevent any conclusive conclusions from being established.

## Conclusion

This meta-analysis quantified a positive correlation between the WMH volume of CSVD and decreased cognition. WMH appears to increase the risk of cognitive impairment in patients with CSVD, and the cognitive decline is a chronic process, whereby WMH predicted the rate of cognitive decline in CSVD for at least 5 years. The results of this meta-analysis suggest that early intervention may prevent or delay the onset of clinical dementia, and minimize cognitive decline for at-risk patients with WMH.

## Data availability statement

The datasets presented in this study can be found in online repositories. The names of the repository/repositories and accession number(s) can be found in the article/supplementary material.

## Author contributions

WG drew up the protocol, performed the search, extracted the data, did the meta-analysis, and drafted the manuscript. JS participated in data extraction, checking, editing and looking overall responsibility for the article, and supervised the management. All the authors were reviewed before the submission of the manuscript.

## Funding

This study was supported in part by the National Key Research and Development Program of China (Grant No. 2018YFC1704100), the National Natural Science Foundation of China (Grant No. 82074362), and the Fundamental Research Funds for the Central Universities (Grant No. 2019-JYB-TD-007).

## Conflict of interest

The authors declare that the research was conducted in the absence of any commercial or financial relationships that could be construed as a potential conflict of interest.

## Publisher's note

All claims expressed in this article are solely those of the authors and do not necessarily represent those of their affiliated organizations, or those of the publisher, the editors and the reviewers. Any product that may be evaluated in this article, or claim that may be made by its manufacturer, is not guaranteed or endorsed by the publisher.
